# Neurovascular coupling, cognition, and cardiac function in stroke-free atrial fibrillation

**DOI:** 10.1016/j.nicl.2025.103932

**Published:** 2025-12-17

**Authors:** Songhong Yue, Jintao Wang, Jiahao Yan, Wanjun Hu, Jun Wang, Yucheng Ding, Laiyang Ma, Pengfei Wang, Na Han, Yurong Ma, Jing Zhang

**Affiliations:** a Department of Magnetic Resonance, Lanzhou University Second Hospital, Lanzhou 730030, China; b Second Clinical School, Lanzhou University, Lanzhou 730030, China; c Gansu Province Clinical Research Center for Functional and Molecular Imaging, Lanzhou, China

**Keywords:** Atrial Fibrillation (AF), Neurovascular Coupling, Cognitive Impairment, Functional magnetic resonance imaging, Echocardiography

## Abstract

**Objectives:**

Atrial fibrillation (AF) is linked to cognitive impairment even without overt stroke, potentially via neurovascular coupling (NVC) dysfunction. We aimed to determine whether alterations in NVC mediate the relationship between cardiac structural remodeling and cognitive deficits in stroke‐free AF (sfAF) patients.

**Methods:**

This prospective cohort study evaluated participants who underwent MR brain scans, transthoracic echocardiography, and completed neuropsychological assessments. We used a neurovascular coupling approach, combining fMRI metrics (amplitude of low-frequency fluctuations (ALFF), fractional ALFF (fALFF), regional homogeneity (ReHo), and degree centrality (DC)) with regional cerebral blood flow maps. Mediation analysis were conducted to explore the relationships among NVC metrics, cardiac parameters, and cognitive performance in sfAF.

**Results:**

Compared with healthy controls, the sfAF patients exhibited significant cardiac remodeling, marked by increased LA and LVEDd and decreased LVEF, alongside poorer cognitive performance on AVLT-Immediate and MoCA. They also showed widespread disruptions in neurovascular coupling, with notably reduced fALFF-CBF coupling in the orbital inferior frontal gyrus. Moreover, this reduction in fALFF-CBF coupling mediated the relationship between elevated LVEDd and short-term memory impairment.

**Conclusion:**

NVC dysfunction mediates the adverse effects of cardiac remodeling on cognitive function in sfAF, supporting a heart-brain axis model as a potential target for early intervention.

## Highlights

• sfAF shows disrupted neurovascular coupling.
• Cardiac remodeling tracks poorer cognition.
• ASL-based perfusion enables simple NVC metrics.
• Orbitofrontal NVC mediates LVEDd–memory link.
• NVC metrics may serve as early biomarkers.

## Graphical abstract

**Figure:**
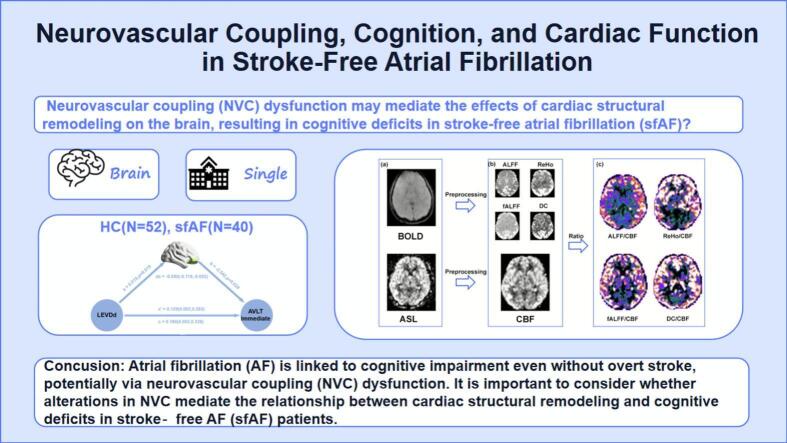

## 1 Introduction

Atrial Fibrillation (AF), the most common cardiac arrhythmia, is a significant risk factor for cognitive dysfunction and dementia, independent of overt ischemic stroke (Nishtala et al., 2018, Liu et al., 2019, Walsh et al., 2024). Even in stroke-free AF (sfAF), patients exhibit cognitive impairment, suggesting that nonembolic pathways may directly compromise brain function (Marzona et al., 2012, Kalantarian et al., 2013). While cardiac remodeling and functional impairment in AF are known to be associated with a higher risk of cognitive decline (Tang et al., 2023), the precise pathophysiological mechanisms linking cardiac dysfunction to cognitive deficits in the sfAF population remain largely unclear, representing a critical gap in our understanding of the “heart-brain interaction”.

To investigate the mechanism of AF-related brain injury, recent focus has shifted to cerebral hemodynamics (Ganesan et al., 2016, York et al., 2018, Hamatani et al., 2023, Al-Khatib, 2023). The beat-to-beat variability in cardiac output inherent to AF can induce chronic cerebral hypoperfusion (Anselmino et al., 2016). Such sustained hypoperfusion is hypothesized to compromise neurovascular coupling (NVC) (Junejo et al., 2020, Scarsoglio et al., 2017). NVC refers to the tight spatial and temporal linkage between neuronal activity and local cerebral blood flow (CBF) changes that is orchestrated by the neurovascular unit, including neurons, astrocytes, endothelial cells, and vascular smooth muscle (Iadecola, 2017). Preclinical models have demonstrated that disrupting NVC leads to oxidative stress, blood–brain barrier breakdown, and synaptic damage, ultimately impairing cognitive performance (Junejo et al., 2020, Graves and Baker, 2020). This positions NVC as a key potential intermediary between cardiac disease and neuronal dysfunction.

Despite its conceptual clarity, quantifying NVC in vivo remains methodologically challenging (Iadecola, 2017). Traditionally, studies of the heart–brain axis in AF have assessed cardiac function (e.g., left ventricular end-diastolic diameter (LVEDd), left ventricular ejection fraction (LVEF)) and brain function (e.g., standard resting-state functional magnetic resonance imaging (rs-fMRI) metrics) in isolation (Nishtala et al., 2018, Liu et al., 2019, Marzona et al., 2012, Tang et al., 2023). Such approaches fail to capture the dynamic interplay between cardiac-induced hemodynamic fluctuations and the brain’s moment-to-moment regulation of perfusion (Junejo et al., 2020, Anselmino et al., 2016, Junejo et al., 2020, Scarsoglio et al., 2017). To address this limitation, rs-fMRI combined with arterial spin labeling (ASL) offers a powerful framework for this purpose. By correlating spontaneous neural activity metrics (from the blood oxygenation level–dependent (BOLD) signal) (Zou et al., 2008, Zang et al., 2007, Zang et al., 2004, Buckner et al., 2009) with simultaneously measured cerebral blood flow (CBF), this multimodal approach yields continuous NVC coefficients that directly quantify neurovascular signaling efficiency (Zhang et al., 2021, Li et al., 2023, Li et al., 2012). Although this BOLD-CBF coupling framework has been validated in aging and other conditions (Boscolo Galazzo et al., 2014, Whittaker et al., 2016, Champagne et al., 2022), to date, no study has applied it to the sfAF population to investigate how cardiac-induced hemodynamic changes might lead to NVC dysfunction and subsequent cognitive impairment.

In this prospective study, we combined cardiac assessments with this advanced rs-fMRI/ASL neuroimaging framework. Considering the proposed role of NVC as a critical intermediary, we hypothesized that sfAF patients would exhibit significant NVC dysfunction and that the degree of this dysfunction would mediate the relationship between cardiac abnormalities and cognitive decline. Therefore, we aimed to: (1) determine whether NVC dysfunction exists in sfAF patients compared to healthy controls; (2) delineate the associations among cardiac-remodeling indices, NVC efficiency, and cognitive performance; and (3) formally test the mediating role of NVC in the pathway from cardiac remodeling to cognitive impairment.

## 2 Materials and methods

### 2.1 Participants

This study adheres to the Declaration of Helsinki and was approved by the relevant Ethics Committee (approval number: 2024A-388). All subjects signed informed consent forms. From January 2024 to January 2025, 40 patients with sfAF and 52 healthy controls (HCs) were recruited. HCs were prospectively enrolled with the aim of achieving a similar distribution of age, sex, and years of education as in the sfAF group. At baseline, there were no significant between-group differences in age, sex, education level, systolic blood pressure, smoking status, or blood cholesterol (all *p* > 0.05; see Table 1).

**Table 1:** Demographic and clinical characteristics of all subjects.

**Variable**	**HC (n = 52)**	**sfAF (n = 40)**	**t/χ^2^**	***P***
			**value**	**value**
**Male, n (%)**	17 (32.70)	19 (47.50)	2.08^b^	0.20
**Age (year)**	60.21 ± 6.05	59.30 ± 8.67	0.57^a^	0.57
**Education (year)**	11.19 ± 2.29	10.93 ± 2.81	0.49^a^	0.63
**CHA**2DS2-VASc score	1.58 ± 0.80	2.03 ± 1.78	−1.48^a^	0.14
**Hypertension, n (%)**	24 (46.20)	21 (40.30)	0.16^b^	0.69
**Diabetes, n (%)**	8 (15.40)	4 (10.00)	0.20^b^	0.65
**HDL (mmol/L)**	1.20 ± 0.39	1.14 ± 0.46	0.67^a^	0.51
**LDL (mmol/L)**	2.63 ± 0.99	2.42 ± 0.89	1.11^a^	0.27
**TC (mmol/L)**	1.66 ± 0.89	1.71 ± 0.96	−0.27^a^	0.79
**TG (mmol/L)**	4.24 ± 1.21	3.95 ± 1.15	1.18^a^	0.24
**Hb (g/L)**	138.48 ± 16.97	133.38 ± 21.00	1.29^a^	0.20
**FIB (g/L)**	3.10 ± 0.90	2.97 ± 0.49	0.82^a^	0.41
**BUN (mmol/L)**	5.42 ± 2.03	6.09 ± 1.34	−2.77^a^	0.06
**AST (U/L)**	25.81 ± 11.36	30.45 ± 22.47	−1.90^a^	0.24
**ALT (U/L)**	26.42 ± 23.22	30.38 ± 29.82	−1.19^a^	0.49

**Note:** Variables are presented as mean ± standard deviation. Statistical significance was set at *p* < 0.05.

**Abbreviations:** HDL, high-density lipoprotein; LDL, low-density lipoprotein; TC, total cholesterol; TG, triglycerides; Hb, hemoglobin; FIB, fibrinogen; BUN, blood urea nitrogen; AST, aspartate aminotransferase; ALT, alanine aminotransferase.

a: two-sample *t*-test.

b: Chi-square test.

Inclusion criteria for sfAF patients were: (1) a diagnosis of AF confirmed by 12-lead electrocardiography (ECG), with assignment of the International Classification of Diseases, 10th Revision (ICD-10) code I48 (Kotalczyk et al., 2021, Kirchhof et al., 2016); (2) age between 45 and 75 years; and (3) right-handedness. Inclusion criteria for HCs were: (1) no history of cardiovascular or cerebrovascular disease; (2) matched demographic and clinical characteristics with the sfAF group; and (3) right-handedness. Exclusion criteria for all participants included: (1) a history of stroke, transient ischemic attack (TIA), or other neurodegenerative diseases (e.g., Parkinson's or Alzheimer's disease); (2) major depressive disorder, alcoholism, or vitamin deficiencies; (3) severe hepatic, thyroid, or renal dysfunction; (4) traumatic brain injury with loss of consciousness; and (5) any contraindications for MRI scanning.

### 2.2 Clinical and neuropsychological assessments

Neurologists investigated all subjects through structured interviews and clinical examinations to rule out any history or signs of stroke and neurodegenerative diseases. We excluded participants with a history of alcoholism, vitamin deficiencies, severe hepatic, thyroid, or renal dysfunction, traumatic brain injury with loss of consciousness, and major depressive disorder. All patients underwent comprehensive laboratory testing to rule out neurological deficits, dementia, and severe metabolic or systemic dysfunction. The CHA_2_DS_2_-VASc score, which considers factors such as congestive heart failure, hypertension, age ≥ 75 years, diabetes mellitus, stroke or transient ischemic attack, vascular disease, age 65–74 years, and sex category, is used to assess the clinical risk of stroke and thromboembolism in both AF patients and controls. Additionally, all AF patients underwent carotid Doppler ultrasound to exclude severe carotid stenosis (>50 %), and transthoracic echocardiography to assess left ventricular ejection fraction (LVEF), with exclusion of those with LVEF < 40 % (Silva et al., 2021).

Neuropsychological assessments included the Mini-Mental State Examination (MMSE), the Activities of Daily Living Scale (ADL), and the Instrumental Activities of Daily Living Scale (IADL) as screening tools to evaluate cognitive and functional status before study enrollment. The Montreal Cognitive Assessment (MoCA) provides a comprehensive assessment of overall cognitive function, while the Auditory Verbal Learning Test (AVLT) measures short-term and delayed memory. The Digit Span Task (DST) evaluates short-term and working memory, the Clock Drawing Test (CDT) assesses visuospatial abilities, and the Verbal Fluency Test (VFT) examines language proficiency and executive function. Additionally, the Geriatric Depression Scale (GDS) is used to screen for depressive symptoms and mood disturbances.

### 2.3 Echocardiographic assessment

Transthoracic echocardiography was performed using a Philips EPIQ CVx system (version 9.0.3). The long axis of the left ventricle near the sternum was obtained, and a vertical line was drawn from the posterior wall of the distal aorta to the endocardium of the posterior wall of the left atrium. The measured cardiac structural parameters included left atrial diameter (LA), LVEDd, left ventricular end-systolic diameter (LVEDs), left ventricular end-diastolic volume (EDV), and left ventricular end-systolic volume (ESV). Apical four-chamber and two-chamber views were acquired, and the LVEF was calculated using the Simpson method. All parameters were assessed over 5–7 consecutive cardiac cycles and averaged to ensure accuracy.

### 2.4 MRI data Acquisition and preprocessing

MRI data were acquired on a 3.0 T Philips CX scanner. Rs-fMRI, T1-weighted, T2-weighted fluid-attenuated inversion-recovery (T2-FLAIR), and 3D-pcASL images were collected with standard protocols. Rs-fMRI preprocessing was performed using DPABI toolbox (DPABI, https://www.rfmri.org/dpabi) in MATLAB R2023a platform (MathWorks, USA) (Yan et al., 2016), including removal of the first 10 volumes, slice-timing correction, Participants with maximum head motion exceeding 3.0 mm in translation or 3.0° in rotation were excluded, all participants in the final cohort met these criteria. The remaining functional images were spatially normalized to MNI space (3 mm isotropic) and visually inspected to ensure registration quality. Nuisance regression was performed using the Friston-24 model (comprising 6 rigid-body parameters, their temporal derivatives, and the squared terms of both), along with white matter and cerebrospinal fluid signals, to rigorously control for motion-related confounds, and smoothing (6 mm full-width at half-maximum (FWHM)). Amplitude of low-frequency fluctuation (ALFF), fractional ALFF (fALFF), regional homogeneity (ReHo), and degree centrality (DC) were computed and Z-scored.

CBF maps were generated by the scanner’s vendor software and preprocessed with SPM12 (https://www.fil.ion.ucl.ac.uk/spm/software/spm12): realignment, co-registration to T1, segmentation, DARTEL-based normalization to MNI space, gray matter (GM) masking, Z-score transformation, and 6 mm smoothing (Ashburner and Friston, 2005, Ashburner, 2007). A detailed description of the fMRI data analysis can be found in the Supplementary material.

### 2.5 NVC analysis

**Fig. 1:**
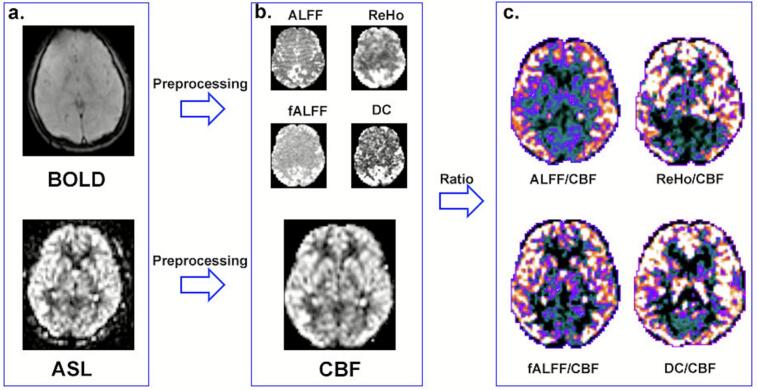
Functional–perfusion processing and ratio mapping workflow. (a) Raw BOLD (top) and ASL (bottom) images and their preprocessing; (b) Preprocessed BOLD‑derived metrics—ALFF, fALFF, ReHo and DC, and ASL‑derived CBF map; (c) Voxel‑wise ratio maps of each functional metric to CBF: ALFF/CBF, ReHo/CBF, fALFF/CBF and DC/CBF. Abbreviations: ALFF, amplitude of low‑frequency fluctuations; fALFF, fractional ALFF; ReHo, regional homogeneity; DC, degree centrality; CBF, cerebral blood flow.

NVC was assessed at whole-brain level using custom scripts incorporating functions from the Multimodal Image Coupling Analysis (MICA) toolkit (Hu et al., 2021). To derive a global measure of NVC, a whole-brain analysis was conducted by calculating the Pearson correlation coefficient between the spatial map of each neural metric (ALFF, fALFF, ReHo, and DC) and CBF map across all gray matter voxels. This procedure yielded four distinct whole-brain NVC coefficients (ALFF-CBF, fALFF-CBF, ReHo-CBF, and DC-CBF) for each participant. For regional NVC assessment, voxel-wise ratios of each neural metric to CBF (ALFF/CBF, fALFF/CBF, ReHo/CBF, and DC/CBF) were computed to reflect local neurovascular balance, generating four parametric NVC ratio maps. These maps were then normalized by dividing each voxel’s value by the subject-specific global mean of the corresponding ratio across gray matter, to improve data normality and reduce inter-subject variability. This normalization was implemented in SPM12 using image arithmetic operations. The resulting maps were used in subsequent group-level analyses (Zhang et al., 2021, Li et al., 2012, Panigrahy et al., 2023). The flowchart for calculating NVC coupling is depicted in Fig. 1.

### 2.6 Statistical analysis

In the statistical analysis, descriptive statistics of demographic and clinical characteristics were first conducted using SPSS 27.0 (IBM Corp., Armonk, NY, USA). Group comparisons of demographic and clinical variables were performed using two-sample t-tests and chi-square tests. For the comparison of NVC patterns at the whole-brain gray matter level, an analysis of covariance (ANCOVA) was applied and Bonferroni correction (*p* < 0.05/5, *p* < 0.01) was used to account for the four NVC metrics (Li et al., 2023). For voxel-wise comparisons of regional NVC ratio maps between HCs and sfAF patients, we used the Statistical Analysis module in DPABI, which implements a univariate general linear model at each voxel. Specifically, group (HC vs. sfAF) was entered as the fixed factor in an ANCOVA, with age, sex, and years of education included as nuisance covariates. The resulting statistical maps were thresholded using Gaussian random field (GRF) correction with a voxel-wise threshold of *p* < 0.001 and a cluster-wise threshold of *p* < 0.05 (two-tailed).

Finally, to further explore the influence of cardiac structure on NVC mode and cognitive function, a multivariate partial least squares correlation (PLSC) analysis was designed to screen out potential variables, with salience-level multiple comparisons controlled using false discovery rate (FDR) correction. Then, a mediating analysis was performed to evaluate whether the NVC mode mediated between cardiac indicators and cognitive impairment. The significance of the mediating effect was verified by the Bootstrap method and 5000 permutation tests (*p* < 0.05).

## 3 Results

### 3.1 Demographic and clinical results

A total of 40 sfAF patients (19 males and 21 females) and 52 HCs (17 males and 35 females) were included in this study (Table 1). There were no significant differences in age (*p* = 0.58), sex (*p* = 0.20), and education level (*p* = 0.63) between the two groups.

### 3.2 Cardiac function and neuropsychological assessment analysis results

Cardiac and cognitive characteristics results are summarized in Table 2. Cardiac function assessment in patients with atrial fibrillation showed that the LA, LVEDd, and LVEDs were increased (*p* < 0.05). Still, the LVEF was decreased (*p* < 0.05), and there was no significant difference in the rate of shortening of the short axis of the left ventricle FS. The cognitive function test results showed that the performance of patients with sfAF in executive function, memory ability and attention was lower than that of HCs. Specifically, the scores of patients with sfAF on MoCA, MMSE, AVLT, and other tests were lower than those in HCs (*p* < 0.001). In addition, there was a decrease in the task performance of working memory and executive function, especially in processing speed and interference inhibition (*p* < 0.05).

**Table 2:** Cardiac and cognitive characteristics of all subjects.

**Variable**	**HC (n = 52)**	**sfAF (n = 40)**	**t value**	***P* value**
**LA**	31.35 ± 2.58	39.67 ± 6.70	−7.45	<0.01**
**LVEF, %**	64.69 ± 1.54	60.71 ± 9.82	2.55	0.02*
**LVEDs**	28.38 ± 2.96	32.63 ± 5.84	−4.20	<0.01**
**LVEDd**	43.25 ± 3.41	47.87 ± 4.86	−5.13	<0.01**
**EDV**	92.35 ± 14.98	108.03 ± 26.05	−3.40	<0.01*
**ESV**	32.52 ± 5.63	43.59 ± 19.74	−3.44	<0.01*
**FS, %**	33.56 ± 2.00	32.83 ± 6.94	0.65	0.52
**ADL**	8.08 ± 0.27	8.12 ± 0.33	−0.74	0.46
**IADL**	12.19 ± 0.49	12.66 ± 1.30	−2.13	0.04*
**GDS**	2.63 ± 2.60	3.03 ± 2.91	−0.70	0.51
**MMSE**	28.08 ± 0.97	24.07 ± 2.02	11.56	<0.01**
**MoCA**	26.63 ± 1.00	20.52 ± 3.12	11.96	<0.01**
**CDT**	3.71 ± 0.61	3.00 ± 0.99	7.08	<0.01**
**VFT**	17.63 ± 3.56	13.78 ± 3.59	8.61	<0.01**
**DST**	11.88 ± 1.48	9.66 ± 1.55	8.60	<0.01**
**AVLT-Immediate**	6.34 ± 1.18	4.10 ± 1.71	5.52	<0.01**
**AVLT-Interference**	6.28 ± 1.25	3.66 ± 1.58	4.02	<0.01**
**AVLT-Delayed**	6.14 ± 1.21	3.48 ± 1.65	5.13	<0.01**
**AVLT-Recognition**	20.78 ± 1.79	18.46 ± 2.16	6.97	<0.01**

**Note:** Variables are expressed as mean ± standard deviations; There was a difference at the level of *p* < 0.05. * means *p* < 0.05 and ** means *p* < 0.01.

**Abbreviations**: LA, left atrial diameter; LVEF, left ventricular ejection fraction; LVEDs, left ventricular systolic diameter; LVEDd, left ventricular end-diastolic diameter; EDV, left ventricular end-diastolic volume; ESV, left ventricular end-systolic volume; FS, rate of short-axis shortening of the left ventricle; ADL, ability of daily living scale; IADL, Instrumental Scale of Activities of Daily Living; CDT, Clock Drawing Test; VFT, Speech Fluency Test; GDS, Geriatric Depression Scale; MMSE, Brief Mental State Examination Scale; MoCA, Montreal Cognitive Assessment Scale; DST, Digital Span Task; AVLT, Auditory Words Learning Test.

### 3.3 ALFF, fALFF, ReHo, DC, and CBF comparison

**Fig. 2:**
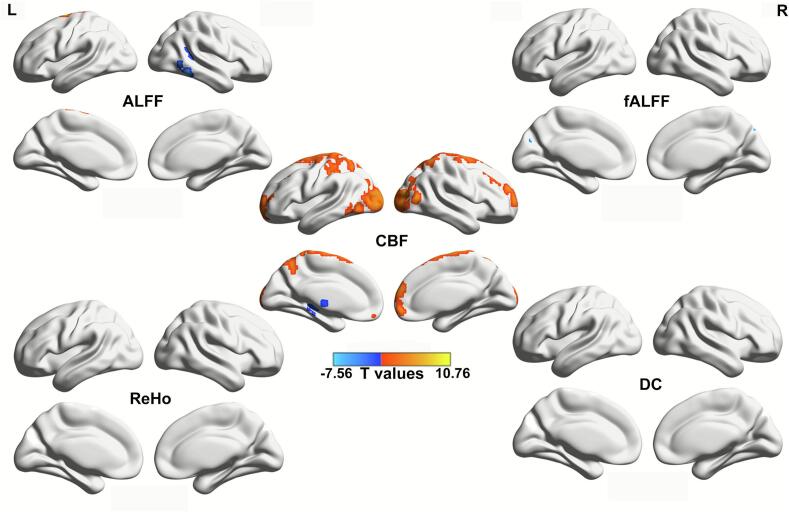
Between-group difference of ALFF, fALFF, ReHo, DC, and CBF maps (voxel *p* < 0.001, cluster *p* < 0.05, GFR corrected).

Compared with HCs, sfAF patients showed reduced CBF across extensive frontal, parietal, and occipital regions, alongside increased CBF in the bilateral thalami. ALFF and fALFF values in the left cuneus and supplementary motor area were reduced, whereas ALFF values in the right inferior temporal gyrus were increased. No significant between-group differences were observed in ReHo or DC. (Fig. 2 and Table 3).

**Table 3:** Brain areas with intergroup differences in ALFF, fALFF, ReHo, DC, and CBF indices (voxel p < 0.001, cluster p < 0.05, GRF corrected).

	**Cortical areas**	**MNI coordinates (mm)**			**Voxels**	**Peak intensity**
		**x**	**y**	**z**		
ALFF	Temporal_Inf_R	48	−42	−6	144	−4.34
	Supp_Motor_Area_L	−9	−6	78	146	3.82
fALFF	Cuneus_L	−6	−78	30	92	4.37
	Supp_Motor_Area_L	−3	6	75	255	4.22
CBF	Thalamus_L	−9	−12	6	233	−6.13
	Thalamus_R	15	−12	6	161	−7.35
	Frontal_Sup_L	−21	60	3	70	4.43
	Frontal_Sup_Medial_R	6	57	18	177	5.40
	Occipital_Mid_L	−21	−99	9	675	5.46
	Paracentral_Lobule_L	−12	−21	78	3185	10.68

### 3.4 Between-group differences in NVC coefficients

**Fig. 3:**
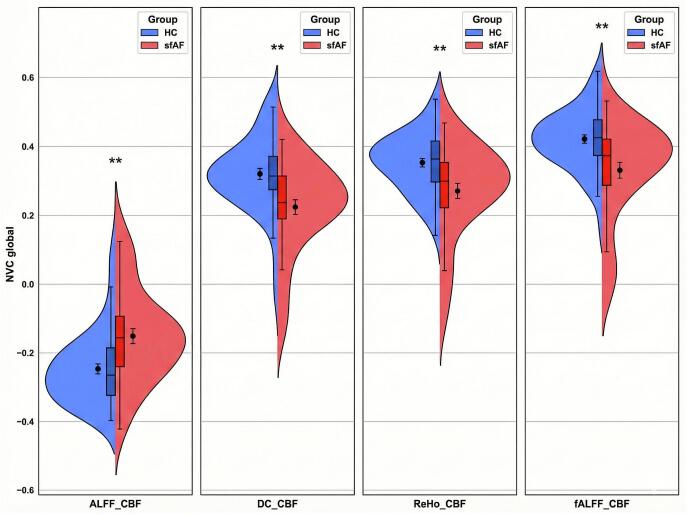
Between-group differences in four NVC patterns at the global GM level (**: Bonferroni-corrected *p* < 0.01; ***: Bonferroni-corrected *p* < 0.001).

At the whole-brain GM level, the two groups showed differences in the following NVC coefficients: ALFF-CBF coefficients (HC, −0.25 ± 0.11; sfAF, −0.15 ± 0.14, *p* < 0.01), fALFF-CBF coefficients (HC, 0.42 ± 0.09; sfAF, 0.33 ± 0.141, *p* < 0.01), ReHo-CBF coefficients (HC, 0.35 ± 0.09; sfAF, 0.27 ± 0.13, *p* < 0.01) and DC-CBF coefficients (HC, 0.32 ± 0.11; sfAF, 0.22 ± 0.13, *p* < 0.01) (Bonferroni correction, *p* < 0.01) (Fig. 3).

**Fig. 4:**
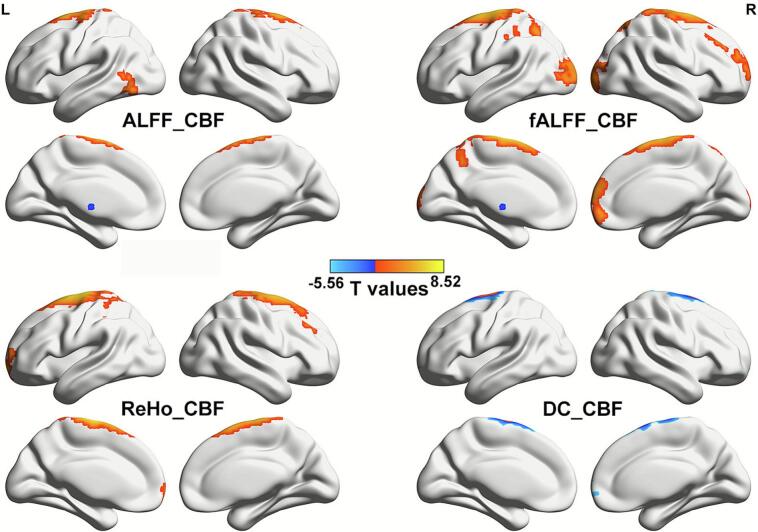
Spatial maps of between-group differences in four NVC patterns at the AAL atlas level (voxel *p* < 0.001, cluster *p* < 0.05, GFR corrected). Red: HCs increased NVC; Blue: HCs decreased NVC. (For interpretation of the references to colour in this figure legend, the reader is referred to the web version of this article.)

At the brain regional level, the NVC coupling comparison showed that, in sfAF patients, the NVC coefficients in the bilateral thalami were increased (for ALFF/CBF, fALFF/CBF, and ReHo/CBF); the NVC coefficients in the left middle occipital gyrus and right paracentral lobule were decreased (ALFF/CBF); the NVC coefficients in the bilateral superior parietal lobules, left middle occipital gyrus, postcentral gyrus, paracentral lobule, and right medial orbital frontal cortex, middle frontal gyrus, and cuneus were decreased (fALFF/CBF); the NVC coefficients in the left superior orbital frontal cortex and superior frontal gyrus were decreased (ReHo/CBF); and the NVC coefficients in the right medial orbital frontal cortex and left superior frontal gyrus were decreased (DC/CBF) (voxel *p* < 0.001, cluster *p* < 0.05, GRF corrected) (Fig. 4). The specific brain regions and statistical values are summarized in Table 4.

**Table 4:** Brain regions with differences in four NVC indices (voxel p < 0.001, cluster p < 0.05, GRF corrected).

	**Cortical areas**	**MNI coordinates (mm)**			**Voxels**	**Peak intensity**
		**x**	**y**	**z**		
ALFF/CBF	Occipital_Mid_L	−42	−63	3	71	4.12
	Paracentral_Lobule_R	9	−21	78	974	8.52
	Thalamus_L	−9	−12	6	74	−4.64
	Thalamus_R	15	−12	6	140	−5.57
fALFF/CBF	Frontal_Mid_R	33	30	45	44	4.41
	Postcentral_L	−27	−39	54	54	4.41
	Cuneus_R	18	−102	6	101	4.59
	Occipital_Mid_L	−21	−99	6	226	5.00
	Parietal_Sup_R	15	−69	63	70	5.19
	Frontal_Med_Orb_R	6	57	−3	159	5.25
	Parietal_Sup_L	−30	−60	51	62	5.28
	Paracentral_Lobule_L	−12	−21	78	1487	10.49
	Thalamus_L	−9	−12	6	95	−5.76
	Thalamus_R	15	−12	−6	108	−7.20
ReHo/CBF	Frontal_Sup_Orb_L	−15	66	−3	70	5.10
	Frontal_Sup_L	−15	−9	72	1638	9.59
	Thalamus_L	−9	−12	4	57	−4.62
	Thalamus_R	15	−12	3	62	−5.95
DC/CBF	Frontal_Med_Orb_R	12	66	−6	140	5.12
	Frontal_Sup_L	−15	−6	72	1162	9.19

### 3.5 Multivariate PLSC analysis

#### 3.5.1 Multivariate PLSC between altered NVC patterns and cardiac function measures

**Fig. 5:**
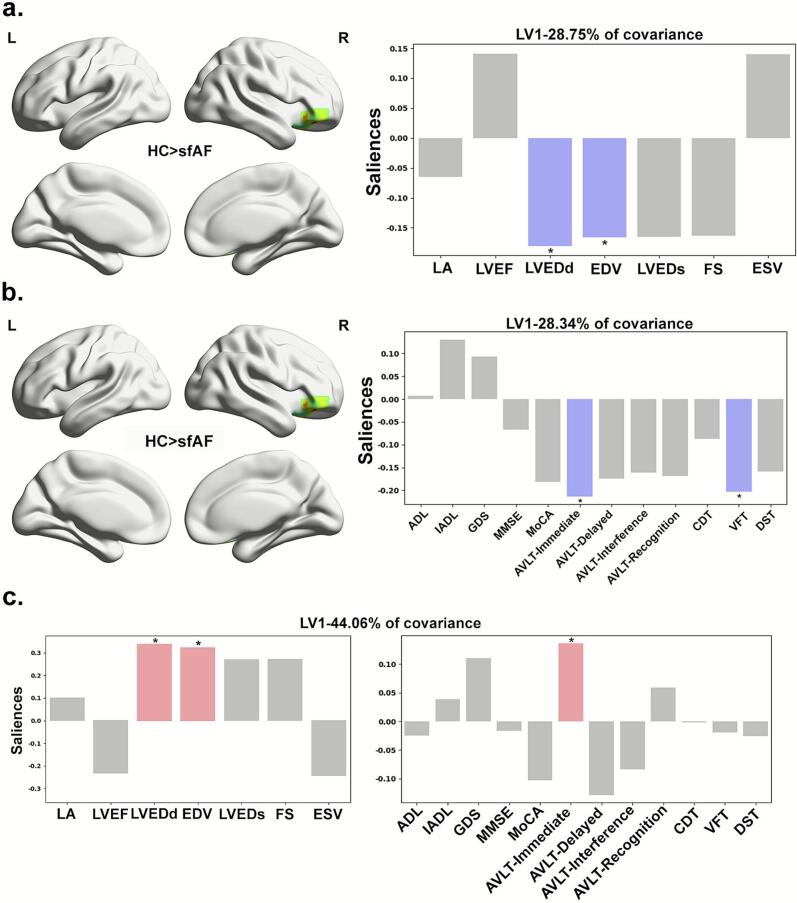
Multivariate plsc analysis linking neurovascular coupling, cardiac function, and cognitive performance in sfAF. Abbreviations: LA, left atrial diameter; LVEF, left ventricular ejection fraction; LVEDs, left ventricular systolic diameter; LVEDd, left ventricular end-diastolic diameter; EDV, left ventricular end-diastolic volume; ESV, left ventricular end-systolic volume; FS, rate of short-axis shortening of the left ventricle; ADL, ability of daily living scale; IADL, Instrumental Scale of Activities of Daily Living; CDT, Clock Drawing Test; VFT, Speech Fluency Test; GDS, Geriatric Depression Scale; MMSE, Brief Mental State Examination Scale; MoCA, Montreal Cognitive Assessment Scale; DST, Digital Span Task; AVLT, Auditory Words Learning Test.

Multivariate PLSC analysis in which changes in NVC patterns were associated with cardiac function measures revealed a latent variable (LV) (FDR correction, *p* < 0.05), resulting in a lower fALFF-CBF coefficient in the right inferior orbital frontal gyrus (ORBinf.R) and higher LVEDd and EDV (Fig. 5a).

#### 3.5.2 Multivariate PLSC between altered NVC patterns and cognitive variables

Multivariate PLSC analysis in which alterations in NVC patterns were associated with neuropsychological measures showed the presence of a LV (FDR correction, *p* < 0.05) that resulted in a lower fALFF-CBF coefficient in the ORBinf.R and a lower short-term memory function score (lower AVLT-Immediate and VFT) in sfAF patients (Fig. 5b).

#### 3.5.3 Multivariate PLSC between cardiac function measures and cognitive variables

Multivariate PLSC analysis linking cardiac function measures with cognitive variables revealed a significant LV (FDR correction, *p* < 0.05). This LV showed that increased LVEDd and elevated EDV were positively correlated with poorer short-term memory performance (lower AVLT-Immediate) in sfAF patients (Fig. 5c).

### 3.6 Mediation analysis

**Fig. 6:**
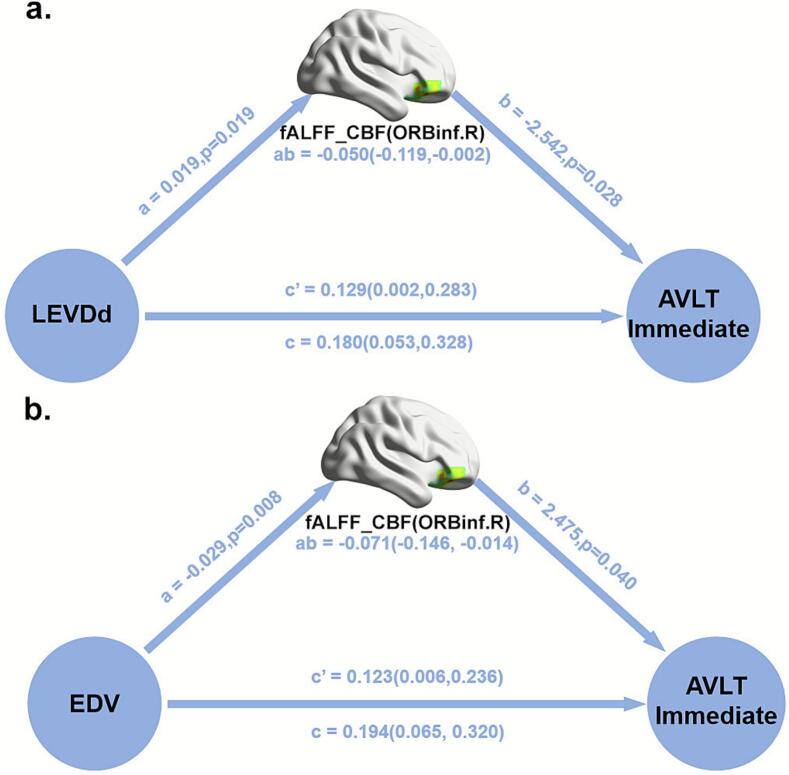
Mediation analysis: right orbital inferior frontal gyrus fALFF-CBF mediates cardiac function and short-term memory in sfAF. Abbreviations: LVEDd, left ventricular end-diastolic diameter; EDV, left ventricular end-diastolic volume; AVLT, Auditory Words Learning Test; ORBinf.R, right orbital inferior frontal gyrus.

To examine the relationship between changes in NVC patterns, cardiac function measures, and cognitive variables in patients with sfAF, we performed mediation analysis to determine whether changes in NVC patterns can mediate the role of clinical markers in cognitive impairment. According to the results of the PLSC analysis, the independent variables were LVEDd and EDV, the dependent variables were the indicators of short-term memory function reflected by the lower AVLT-Immediate score, and the fALFF-CBF coefficient in the right orbital inferior frontal gyrus as a proposed mediator based on the PLSC results. Mediation analysis showed that the lower fALFF-CBF coefficient in the ORBinf.R fully mediated the effect of LVEDd (c′ = 0.129, [95 %CI(0.002, 0.283)]) and EDV (c′ = 0.123, [95 %CI (0.006, 0.236)]) on short-term memory deficits in patients with sfAF (Fig. 6).

## 4 Discussion and Conclusions

This study investigated the association between cardiac structural and functional abnormalities and cognitive impairment and the mediating role of NVC dysregulation in sfAF patients. We found that: (1) sfAF patients exhibited significant NVC impairment, characterized by globally reduced coupling between multiple neural activity metrics and cerebral blood flow, alongside a pattern of widespread cortical hypoperfusion, (2) the severity of adverse cardiac remodeling, particularly increased LVEDd and EDV, was significantly associated with both this NVC inefficiency and poorer performance across cognitive domains like short-term memory and executive function, and (3) critically, NVC dysregulation specifically within the right inferior frontal gyrus statistically mediated the pathway from cardiac remodeling to short-term memory deficits. The results provide new multimodal evidence for a deeper understanding of the heart-brain interaction of sfAF, and further support an important role of NVC in maintaining cognitive function.

We found weak across-voxel correlations between CBF and four parameters representing spontaneous neural activity in HC and sfAF patients at global gray matter level, and the ALFF-CBF coupling did not become more negative; rather, its absolute magnitude decreased (shifted toward zero), while the other three NVC couplings likewise weakened. In other words, the common feature across all four global NVC couplings is an overall contraction of coupling strength toward zero—indicative of poorer supply–demand matching—rather than a contradiction in correlation direction. The results may be due to: First, metric definition and scaling: ALFF reflects absolute low-frequency power, whereas fALFF (proportion of ALFF to total amplitude), ReHo (consistency of activities within the cluster), and DC (summation of functional connections for a given voxel including short and long range functional mapping) capture distinct BOLD properties (Zuo et al., 2012, Zou et al., 2008, Zang et al., 2007, Zang et al., 2004); combined with GM-wise Z-standardization and typical smoothing, this can yield a negative ALFF-CBF spatial correlation in healthy cortex while the other three remain positive, such that the key disease signal is the common attenuation toward zero rather than sign reversal. Second, quantification biases inherent to the 3D-pcASL sequence used to derive our CBF maps (partial-volume effects and arterial transit-time differences) can underestimate cortical CBF and overestimate deep-gray CBF, distorting CBF-BOLD spatial covariance and globally weakening couplings (Chappell et al., 2021). Third, the hemodynamic burden of AF, characterized by beat-to-beat output variability and reduced vascular reactivity and autoregulation (Junejo et al., 2019), likely weakens the correspondence between resting neural properties (amplitude, synchrony, topology) and basal perfusion, leading to the systemwide reduction in coupling strength observed across all four NVC readouts.

Compared to the BOLD-CBF correlations that reflect a comprehensive change at the global level, the BOLD/CBF ratios were used to quantify the amount of blood flow per unit of functional segregation and integration and were understood as proxies of regional NVC to provide more details about NVC (Iadecola, 2017, Hu et al., 2021). sfAF patients showed not only a global decline on general screening tools (MoCA and MMSE), but also disproportionate impairments in short-term memory, working memory, and executive control, particularly in processing speed and interference inhibition. The spatial pattern of regional NVC abnormalities closely mirrored this cognitive profile: widespread cortical hypoperfusion in frontal, parietal, and occipital regions was accompanied by reduced NVC coefficients in prefrontal, orbitofrontal, parietal, and occipital cortices, despite largely preserved ReHo and DC magnitudes. Multivariate PLSC analysis further captured this correspondence in a data-driven manner: one latent variable linked lower fALFF/CBF coefficients in the ORBinf.R with poorer short-term memory performance (lower AVLT-Immediate scores) and reduced verbal fluency (lower VFT scores). This mode of covariation indicates that, within the sfAF cohort, individuals with more pronounced prefrontal NVC “insufficiency” tend to belong to the subgroup with greater frontally mediated cognitive deficits. Given the established role of the prefrontal cortex in strategic encoding, retrieval, and executive control (Buckner et al., 2009, Zuo et al., 2012, Kringelbach, 2005), a persistent “metabolic mismatch”—where hemodynamic supply fails to adequately meet the metabolic demands of spontaneous activity—offers a plausible mechanism linking NVC disruption in these regions to the observed memory and executive dysfunction. Similarly, reduced ALFF/CBF and fALFF/CBF coupling in the superior parietal and middle occipital gyri overlapped with regions supporting visuospatial and attentional processing, in line with the patients’ poorer performance on tasks probing these domains (Talwar et al., 2019, Renier et al., 2010). Although our analysis were not designed to test simple voxelwise correlations, the convergence of group-level differences and multivariate PLSC patterns supports the interpretation that regional NVC failure is a physiologically meaningful contributor to the multidomain cognitive decline observed in sfAF.

We also explored how cardiac remodeling might give rise to the observed neurovascular and cognitive changes. Consistent with established AF pathophysiology, sfAF patients in our cohort showed atrioventricular remodeling with larger LVEDd/EDV and reduced ejection fraction. Our multivariate PLSC together with mediation analysis indicated that individuals with greater ventricular enlargement tended to have lower ORBinf.R fALFF/CBF and poorer short-term memory, with regional NVC in ORBinf.R transmitting part of this association. This covariation pattern aligns with emerging evidence from large-scale cohorts showing that markers of cardiac remodeling, such as left atrial enlargement or left ventricular hypertrophy, are independent predictors of cognitive decline and are particularly related to executive and memory impairment, even in the absence of overt stroke (Zhang et al., 2019, Diener et al., 2019, Singh-Manoux et al., 2017, Rydén et al., 2019). Recent neuroimaging studies in AF further map these cardiac-driven deficits onto frontal and default mode network regions, reporting reduced gray-matter volume and functional disruption in prefrontal and hippocampal areas that parallel the executive and memory profile observed in our sfAF cohort (Graff-Radford et al., 2016, Petersen et al., 2024, Vrinceanu et al., 2022). These converging findings highlight the particular vulnerability of frontally mediated networks to chronic hemodynamic stress. Mechanistically, our results are compatible with the notion that the orbitofrontal and broader prefrontal cortex may act as metabolically demanding hubs that are especially sensitive to unstable perfusion, such that beat-to-beat output variability and ventricular volume overload in sfAF progressively erode the neurovascular unit’s buffering capacity and lead to inefficient coupling between neural activity and basal blood flow (Junejo et al., 2020, Anselmino et al., 2016, Junejo et al., 2020, Scarsoglio et al., 2017). At the same time, LVEDd and EDV remained associated with cognition in our models. Together with established links between AF and covert brain infarcts, white-matter hyperintensities, cerebral small-vessel disease, and systemic inflammation (Junejo et al., 2020, Graves and Baker, 2020, Silva et al., 2021, Diener et al., 2019, Graff-Radford et al., 2016, Petersen et al., 2024, De Vis et al., 2018), this pattern suggests that NVC disruption represents one key, quantifiable pathway within a broader, multimodal heart–brain mechanism rather than a single sufficient explanation.

There were some limitations in this study. First, this study's relatively limited sample size may have led to an underestimation of some subtle effects, and it was not easy to conduct more granular subgroup analysis. Second, the cross-sectional design limited causal inference, and longitudinal follow-up was required to verify whether atrial fibrillation cardiac abnormalities preceded or accompanied the evolution of NVC dysregulation. Third, potential confounding factors such as sleep quality, microinfarction, and drug use were not adequately controlled, which might have affected the cognitive assessment and brain imaging results. It was suggested that future multicenter, large-scale, and prospective studies could be carried out, and that the inclusion of more comprehensive clinical and biological indicators would help to more accurately elucidate the mechanism and intervention strategies of cognitive impairment related to NVC and sfAF.

In conclusion, this study highlights the utility of NVC dysfunction as a key marker of heart-brain axis impairment in sfAF. We observed prevalent NVC dysfunction in patients with sfAF, demonstrating altered cerebrovascular regulation in this condition. Furthermore, our findings revealed a significant mediating effect of NVC dysfunction, particularly in the right orbital inferior frontal gyrus, on the relationship between cardiac remodeling (increased LVEDd) and short-term memory impairment. These results suggest that NVC dysregulation could be a potential mechanism of central nervous system injury in atrial fibrillation and a target for interventions to protect brain function.

## Funding statement

This work was supported by Gansu Province Science and Technology Plan project (No.24YFFA047), by the Natural Science Foundation of Gansu Province (No.24JRRA328), (No.22JR5RA997), by the Lanzhou city technology bureau talent innovative start-ups (No.2022-RC-74).

## CRediT authorship contribution statement

**Songhong Yue:** Writing – review & editing, Writing – original draft, Visualization, Methodology, Funding acquisition, Formal analysis, Conceptualization. **Jintao Wang:** Writing – review & editing, Writing – original draft, Visualization, Methodology, Formal analysis, Conceptualization. **Jiahao Yan:** Writing – review & editing, Methodology, Formal analysis. **Wanjun Hu:** Writing – review & editing, Investigation, Data curation. **Jun Wang:** Writing – review & editing, Writing – original draft, Visualization, Methodology, Formal analysis, Conceptualization. **Yucheng Ding:** Writing – review & editing, Investigation, Data curation. **Laiyang Ma:** Resources, Investigation, Data curation. **Pengfei Wang:** Resources, Investigation, Data curation. **Na Han:** Writing – review & editing, Methodology, Formal analysis. **Yurong Ma:** Writing – review & editing, Methodology, Formal analysis. **Jing Zhang:** Writing – review & editing, Supervision, Resources, Project administration, Funding acquisition, Conceptualization.

## Data Availability

Data will be made available on request.
