# Supplementary material for: Neurovascular coupling, cognition, and cardiac function in stroke-free atrial fibrillation
Source: Neuroimage Clin. 2025 Dec 17;49:103932. doi: 10.1016/j.nicl.2025.103932 (PMC12775862; doi:10.1016/j.nicl.2025.103932)
Supplement: Supplementary Data 1 [file mmc1.docx]

**2.4 MRI Data Acquisition and Preprocessing**

MRI data were acquired using a 3.0T Philips CX Scanner with a 32-channel head coil. During scanning, all participants remained awake with their eyes closed. High-resolution T1-weighted images were obtained using a sagittal magnetization-prepared fast gradient echo sequence with the following parameters: echo time (TE) = 2.99 ms, repetition time (TR) = 8.06 ms, inversion time (TI) = 450 ms, flip angle (FA) = 15°, field of view (FOV) = 300 × 300 mm², matrix = 512 × 512, slice thickness = 1.0 mm, and number of slices = 160. The rs-fMRI data based on BOLD contrast were collected using echo-planar imaging (EPI) with the following parameters: TE = 35 ms, TR = 2000 ms, FA = 90°, slice gap = 0 mm, FOV = 280 × 280 mm², matrix = 128 × 128, slice thickness = 4.0 mm, and number of slices = 40. A background-suppressed 3D-pCASL sequence was acquired with the following parameters: TE = 10.85 ms, TR = 4904 ms, FA = 111°, FOV = 260 × 260 mm², matrix = 128 × 128, slice thickness = 4.0 mm, number of slices = 40, and post-labeling delay = 2000 ms. CBF maps were generated in-line on the scanner using vendor-supplied software. An axial T2-weighted fluid-attenuated inversion-recovery (T2-FLAIR) sequence was also acquired to screen for covert infarcts and white matter hyperintensities. Imaging parameters were: TE = 120 ms, TR = 11000 ms, TI = 2600 ms, FA = 90°, FOV = 240 × 240 mm², matrix = 256 × 256, slice thickness = 5 mm with a 1 mm interslice gap, and number of slices = 30.

The preprocessing of rs-fMRI images, the DPABI toolbox (DPABI, http://www.rfmri.org/dpabi)^1^, based on the MATLAB R2023a platform (MathWorks, USA), was used. The processing flow consists of removing the first 10 time points to ensure signal balance, then timing correction for the remaining time points, and realigning the functional images to correct for head movement (range of motion limited to 3.0 mm and 3.0°). Next, the functional images were normalized to the Montreal Neurological Institute (MNI) template space (3 × 3 × 3 mm³ resolution), and covariates (head movement, cerebrospinal fluid signal, and white matter signal) were removed. Finally, the data were smoothed using a Gaussian filter of 6×6×6 mm full-width at half-maximum (FWHM) to ensure the accuracy of subsequent analysis.

To calculate functional neuroimaging indicators, ALFF, fALFF, ReHo, and DC were extracted. Among them, ALFF calculates the frequency-domain power spectrum of the time series by fast Fourier transform, and extracts the mean of the square root power spectrum of the 0.01-0.1 Hz band. In contrast, fALFF is calculated by the ratio of the root mean square of the ALFF to the full-frequency power spectrum. The DC was calculated by constructing a voxel-level functional network, calculating the Pearson correlation of each voxel with other voxels in the whole brain, and using a threshold of 0.25 to rule out weak correlations. ReHo is calculated based on the Kendall coefficient of agreement (KCC) between the voxel time series and its adjacent 26 voxels. These indicators were normalized and converted to Z-score mappings to eliminate inter-individual differences.

For ASL images, corresponding CBF maps were generated using the vendor-provided image post-processing software (IntelliSpace Portal, Philips Healthcare, Best, The Netherlands). The CBF maps were preprocessed using SPM12 (http://www.fil.ion.ucl.ac.uk/spm/software/spm12). First, all CBF volumes were realigned to correct for residual motion, and realigning the CBF images to correct for head movement (range of motion limited to 3.0 mm and 3.0°). Next, each subject’s CBF series was co‐registered to their high‐resolution T1‐weighted structural image using a six‐parameter rigid‐body transform, ensuring precise alignment of perfusion and anatomy. The T1 image was then segmented into gray matter, white matter, and cerebrospinal fluid tissue classes and spatially normalized to the MNI‐152 template (3 × 3 × 3 mm³) using the unified segmentation algorithm^2^ followed by DARTEL diffeomorphic registration for improved inter‐subject correspondence^3^. The resulting deformation fields were applied to the co‐registered CBF maps to bring them into MNI space. Once normalized, CBF values were Z‐score transformed across all voxels within a GM mask to reduce inter‐individual variability. Finally, the normalized CBF maps were smoothed with a 6 mm FWHM Gaussian kernel to enhance signal‐to‐noise ratio and meet the assumptions of Gaussian random field theory in subsequent voxel‐wise analysis.

**References**

1. Yan C G, Wang X D, Zuo X N, et al. Dpabi: Data processing & analysis for (resting-state) brain imaging. *Neuroinformatics* **14** (3), 339-351 <https://doi.org/10.1007/s12021-016-9299-4> (2016).

2. Ashburner J, Friston K J. Unified segmentation. *Neuroimage* **26** (3), 839-851 <https://doi.org/10.1016/j.neuroimage.2005.02.018> (2005).

3. Ashburner J. A fast diffeomorphic image registration algorithm. *Neuroimage* **38** (1), 95-113 <https://doi.org/10.1016/j.neuroimage.2007.07.007> (2007).
